# The Transient Receptor Potential Vanilloid 1 Is Associated with Active Inflammation in Ulcerative Colitis

**DOI:** 10.1155/2018/6570371

**Published:** 2018-07-29

**Authors:** Joel Jesús Toledo-Mauriño, Janette Furuzawa-Carballeda, Marco A. Villeda-Ramírez, Gabriela Fonseca-Camarillo, Daniela Meza-Guillen, Rafael Barreto-Zúñiga, Jesús K. Yamamoto-Furusho

**Affiliations:** ^1^Inflammatory Bowel Disease Clinic, Department of Gastroenterology, Instituto Nacional de Ciencias Médicas y Nutrición Salvador Zubirán, Mexico City, Mexico; ^2^PECEM (Combined Study Plan in Medicine), Faculty of Medicine, Universidad Nacional Autónoma de México, Mexico City, Mexico; ^3^Department of Immunology and Rheumatology, Instituto Nacional de Ciencias Médicas y Nutrición Salvador Zubirán, Mexico City, Mexico; ^4^Department of Endoscopy, Instituto Nacional de Ciencias Médicas y Nutrición Salvador Zubirán, Mexico City, Mexico

## Abstract

The transient receptor potential vanilloid 1 (TRPV1) may play a role in the pathogenesis of ulcerative colitis (UC). The aim of the study was to determine the gene and protein expression of TRPV1 in UC patients and noninflamed controls. Gene expression was performed by RT-PCR, and protein expression was performed by immunohistochemistry. The gene expression of TRPV1 was significantly increased in the remission UC group compared to active UC patients (*P* = 0.002), and an upregulation of the TRPV1 gene was associated with clinical outcomes such as age at diagnosis (<40 years) (*P* = 0.02) and clinical disease course characterized by relapsing and continuous activity (*P* = 0.07). TRPV1 immunoreactive cells were conspicuously higher in all intestinal layers from active UC patients compared with noninflamed control tissue. These findings suggest that TRPV1 might be involved in UC pathogenesis.

## 1. Introduction

Inflammatory bowel disease (IBD) comprises both conditions Crohn's disease (CD) and ulcerative colitis (UC) which are characterized by chronic and remitting course. It has been speculated that there is abnormal barrier function, inflammatory infiltrates in submucosa and mucosa, and dysregulated cytokine and T-helper cell profiles [1, 2].

The transient receptor potential vanilloid 1 (TRPV1) is a nonselective cation channel which belongs to a family of receptors that have been involved in the permeability for divalent and monovalent cations including Ca^2+^, Na^2+^, and Mg^2+^ named TRP (transient receptor potential) [3, 4].

The capsaicin is one of the natural ligands of TRPV1, a spicy component of hot peppers. Capsaicin suppresses the expression of inflammatory cytokines such as interleukins (IL-6 and IL-8), tumor necrosis factor (TNF-*α*), cyclooxygenase-2 (COX2), and prostaglandin E2 (PGE2) in chronic inflammatory states [5].

Previous studies have demonstrated that TRPV1 is involved in the stimulation of pain and inflammation specifically in those patients with irritable bowel syndrome (IBS) [6].

TRPV1 knockout mice models have reported that overexpression of TRPV1 was associated with enhancement of clinical symptoms and histopathological changes such as neutrophil accumulation [7, 2]. On the other hand, TNBS-induced colitis animal model has shown that TRPV1 may have a protective role in the inflammatory process due to TRPV1 −/− knockout mice, revealing low inflammation activity index and myeloperoxidase activity level [8].

No previous studies have evaluated the role of TRPV1 in UC patients regarding clinical outcomes and the type of TRPV1-expressing cells. The aim of this study was to determine the gene and protein expression in patients with UC and noninflamed controls as well as its association with clinical outcomes.

## 2. Materials and Methods

### 2.1. Study Subjects

A total of 53 individuals were divided into 3 groups: 17 active UC, 17 remission UC, and 19 normal controls without colonic inflammation (non-IBD controls) documented by histology. The diagnosis of UC was performed according to clinical, endoscopic, and histopathological findings.

All UC patients were included during the period from January 2014 to July 2015 belonging to the Inflammatory Bowel Disease Clinic at the National Institute of Medical Sciences and Nutrition Salvador Zubirán. Relevant clinical and demographic information from all UC patients were collected from interview and clinical medical records. The variables evaluated were age at diagnosis, gender, type of medical treatment (5-aminosalicylates, steroids, thiopurines, and biological therapy), disease extension according to Montreal Classification (E3: pancolitis, E2: left colitis, and E1: proctitis), the presence of extraintestinal manifestations (peripheral and axial joint affection, primary sclerosing cholangitis, pyoderma gangrenosum, erythema nodosum, and uveitis) and clinical course classified as initially active and long-term remission, intermittent activity (<2 relapses per year), and chronic continual activity (persistent activity without remission periods). Colonoscopy was performed for taking biopsies and to calculate the Mayo Score Activity Index [9–11]. The normal control group consisted of noninflamed controls without inflammatory bowel disease undergoing colonoscopy for other reasons. All patients who agree to participate in the study signed written informed consent.

### 2.2. Sample Processing and Gene Expression Analysis

All colonic biopsies were taken by colonoscopy and were immediately placed in RNAlater (Ambion, Austin, TX, USA) and stored at −70°C until processing. Total RNA extraction from colonic biopsies was made using RNA extraction kit (High Pure RNA Tissue Kit, Roche). Biopsies were mixed using homogenizer for 1 minute with lysis buffer, one wash with ethanol was made using purification columns, the mix was then centrifuged at 13,000 ×g for 15 seconds, a second wash was made by using washing buffer at 13,000 ×g for 15 seconds, and finally, 100 *μ*L was added to elution buffer in order to dilute total RNA. Electrophoresis confirmed RNA extracts in a 1% agarose gel visualized by a UV transilluminator. The cDNA synthesis was made from 20 *μ*L of total RNA reverse transcription based on the following protocol: preincubation: 25°C × 10 minutes, incubation: 55°C × 30 minutes, and followed by denaturalization: 85°C × 5 minutes by using a thermocycler (Perkin-Elmer).

The gene expression of TRPV1, IL-6, and *β*-actin (reference gene) was performed by real-time polymerase chain reaction (RT-PCR) using 20 *μ*L of cDNA for each gene. Amplification was made by the following conditions: denaturalization program at 95°C × 10 minutes, 45 amplification cycles (95°C × 10 seconds, alignment 60°C × 10 seconds, extension 40°C × 30 seconds), and a cooling cycle at 40°C × 30 seconds. The thermocycler LightCycler 2.0 Roche® was used by employing validated assays for quantification (reproducibility and linearity), with sense and antisense oligonucleotides from Invitrogen® and TaqMan probes for each gene (Universal Probe Library, Library Set, Human, Roche). Oligonucleotide sequences are shown in Table 1. It is important to note that we used IL-6 gene expression level because our group already demonstrated that IL-6 is a better marker than TNF-*α* for detecting inflammation in the colonic mucosa and had a high correlation with histological activity in Mexican patients with UC [12].

### 2.3. Immunohistochemical Procedure

The TRPV1 protein expression was determined by using 5 *μ*m thick sections of available formalin-fixed paraffin-embedded tissue from 5 colon surgical specimens of severe active UC patients and 5 from controls without IBD. All surgical specimens were deparaffinized and rehydrated with xylene and alcohol. Endogenous peroxidase and binding of nonspecific proteins were blocked with 3% H_2_O_2_ and 10% of normal donkey serum (ABC Staining System; Santa Cruz Biotechnology), respectively. All colon surgical specimens were incubated with rabbit anti-human TRPV1 (Santa Cruz Biotechnology) diluted at 10 *μ*g/mL for 18 h at 4°C. Binding was detected by incubating sections for 60 min at room temperature with goat anti-rabbit IgG antibody alkaline phosphatase conjugate (Santa Cruz Biotechnology). Slides were incubated with the substrate permanent red (Sigma-Aldrich Co.) for 10 min. The sections were counterstained with Mayer's hematoxylin (Lillie's modification) (DAKO, Glostrup, Denmark) and immersed into a bath of 7 mM/L ammonia water, rinsed gently in deionized water for 2–5 min, dehydrated, and mounted with Faramount aqueous mounting medium (DAKO). Negative control staining was performed with normal donkey serum diluted 1 : 100, instead of primary antibody, and the IHC universal negative control reagent specifically designed to work with rabbit, mouse, and goat antibodies (IHC universal negative control reagent, Enzo Life Sciences Inc., Farmingdale, NY, USA, ADI-950-231). The reactive blank was incubated with phosphate buffer saline-egg albumin (Sigma-Aldrich) instead of the primary antibody. Controls excluded nonspecific staining or endogenous enzymatic activities. Morphometric evaluation of stained sections was performed in a blinded manner. TRPV1-expressing cells were assessed by estimating the positive staining cells in three fields (×320) and were reported as the percentage of immunoreactive cells of the inflammatory infiltrates located at mucosa, submucosa, muscular, and serosa. Results are expressed as the mean ± standard error of the mean (SEM) of cells quantified by the program Image-Pro Plus version 5.1.1.

### 2.4. Ethical Considerations

This study was performed according to the principles expressed in the Declaration of Helsinki. The study was approved by the Ethical and Medical Committee at the Instituto Nacional de Ciencias Médicas y Nutrición, and a written informed consent was obtained from all individuals.

### 2.5. Statistical Analysis

The statistical analysis was performed using SPSS version 17.0, Kruskal-Wallis nonparametric test, Spearman's correlation, Fisher's exact test, and odds ratio (OR) in order to determine the strength of association. Immunohistochemistry statistical analysis was done by using one-way analysis of variance on ranks by Dunn's method for all pairwise multiple comparison procedure (SigmaStat 11.2 program, Aspire Software International, Leesburg, VA, USA). Data were expressed as median, range, and mean ± standard deviation (SD)/standard error of the mean (SEM). A *P* value < 0.05 was considered as statistically significant.

## 3. Results

### 3.1. Demographic and Clinical Characteristics

A total of 34 patients with UC (17 female and 17 male with a mean age of 40.60 ± 13.38 years) and 19 non-IBD controls (10 female and 9 male with a mean age of 47.22 ± 15.92 years) were studied. The disease extent was evaluated by total colonoscopy, and biopsies were obtained from all segments of the colon. The medical was based on 97.1% with 5-aminosalicylates (5-ASA), 29.4% steroids, 23.5% thiopurines, and 2.9% anti-TNF therapy as shown in Table 2.

### 3.2. TRPV1 and IL-6 Gene Expression in Colonic Tissue from Patients with UC

**Figure 1:**
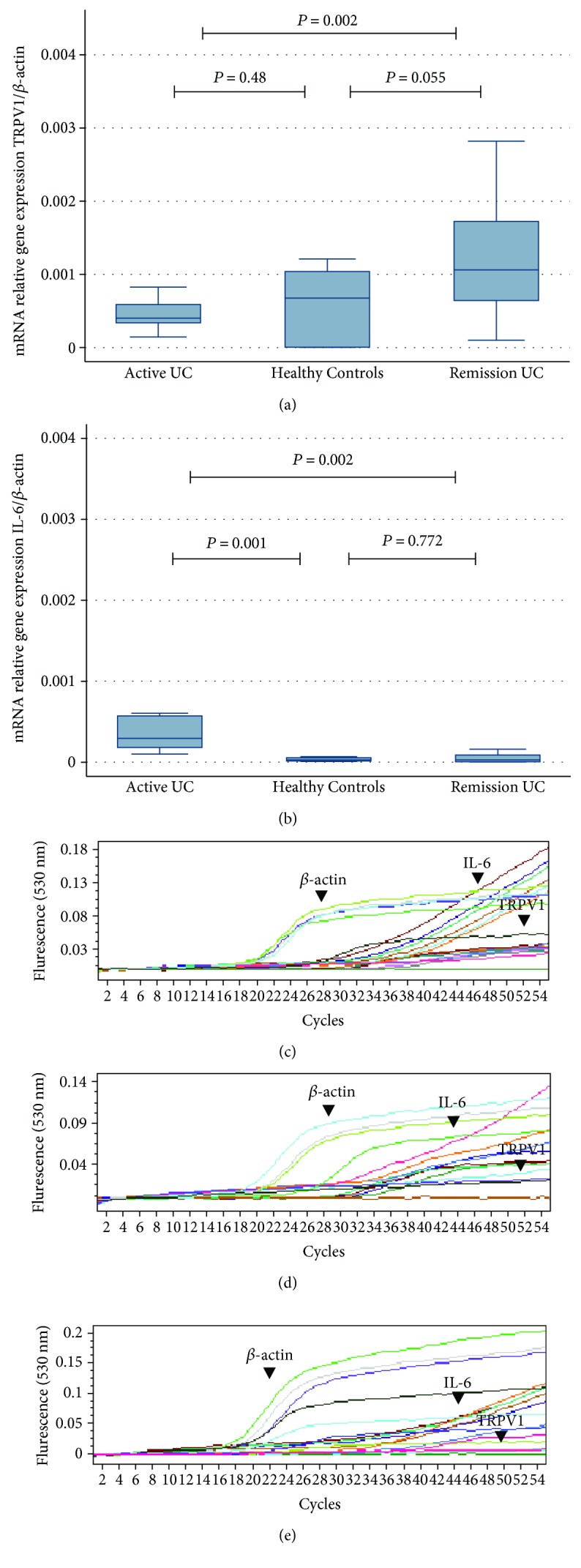
Gene expression of TRPV1 and IL-6 quantified by RT-PCR in colonic mucosa from patients with active and remission UC compared to non-IBD controls. (a) TRPV1 mRNA expression levels. (b) IL-6 mRNA expression levels. Bars show mean ± standard error of the mean of transcript levels from UC patients with *β*-actin as housekeeping gene determined by 2^−∆∆*Ct*^. ^∗^*P* value < 0.05 was considered as significant. Panels (c), (d), and (e) showed original RT-PCR cycles to clearly appreciate the level of TRPV1 mRNA expression in active UC, normal controls, and remission UC, respectively.

The TRPV1 gene expression was significantly increased in the remission UC group compared to the active UC group (*P* = 0.002). The gene expression of TRPV1 was higher in UC remission compared to the normal controls without inflammation (*P* = 0.055). No significant difference was found between patients with active UC compared with normal controls as shown in Figure 1. The TRPV1 downregulation was associated with age at diagnosis younger than 40 years (*P* = 0.02) and clinical disease course characterized by relapsing and continuous activity (*P* = 0.07) as shown in Table 2.

The gene expression of TRPV1 and IL-6 in patients with active UC, remission UC, and normal controls are shown in Figure 1. It is important to note that active UC patients had significantly higher levels of IL-6 gene expression than those in normal controls and remission UC patients (*P* = 0.001 and *P* = 0.002, resp.). The gene expression of IL-6 was similar in normal controls and the remission UC group (*P* = 0.772).

### 3.3. TRPV1-Expressing Cells in Patients with UC and Non-IBD Controls

**Figure 2:**
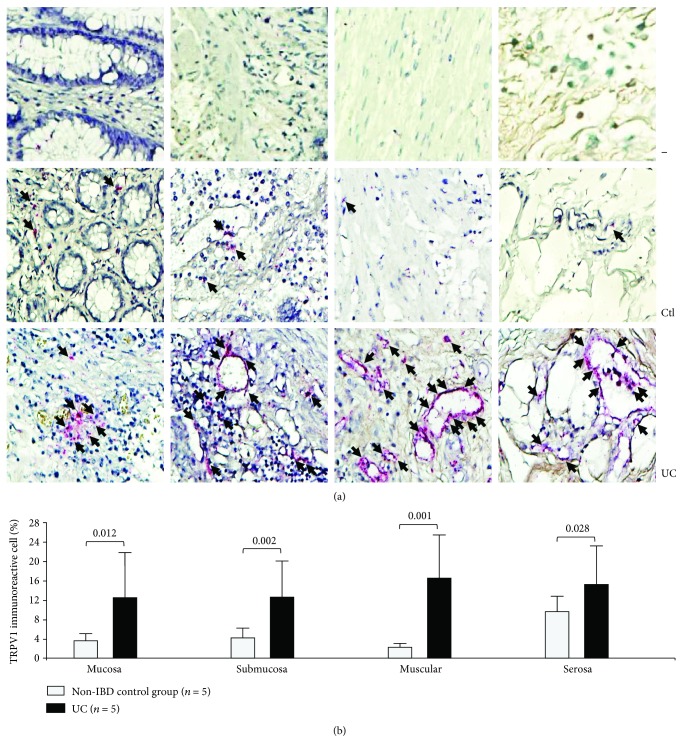
TRPV1 protein expression in colonic tissue from patients with severe ulcerative colitis and controls. Negative controls are also presented (−). (a) Representative immunoperoxidase photomicrographs of ulcerative colitis (lower panel, *n* = 5) and non-IBD colonic tissue (control; upper panel, *n* = 5). Arrows depict TRPV1immunoreactive cells in mucosa, submucosa muscular, and serosa layers. Original magnification was ×320. (b) Bars indicate percentage of TRPV1-producing cells in noninflamed colonic tissues (control, *n* = 5) and active UC patients (*n* = 5). Results are expressed as mean ± standard deviation.

The TRPV1 protein expression showed that percentage of TRPV1 immunoreactive cells was conspicuously higher in lymphocytes, mast, endothelial, epithelial, and muscle cells localized in the mucosa, submucosa, muscular, and adventitia from severe active UC patients compared to normal control colonic tissue as shown in Figure 2.

## 4. Discussion

The findings of the present study demonstrated that there is a differential gene and protein expression of TRPV1 in UC patients compared to normal controls without colonic inflammation. The TRPV1 gene expression was significantly decreased in patients with active UC and was associated with age at diagnosis younger than 40 years and clinical disease course characterized by relapsing and continuous activity.

On the other hand, the protein expression of TRPV1 was increased in several types of cells in all colonic layers from severe active UC patients compared with normal colonic tissue.

This paradoxical finding between the gene and protein expression could be explained because the mRNA presence is not always related to protein expression (correlation between the RNA and protein profile is between 33 and 40% and depends on the half-life of different proteins (minutes to days) and also several mechanisms such as posttranscriptional modification, RNA transport, mRNA degradation, complex gene regulatory process, RNA processing, alternative splicing, and RNA stability). Besides, colonic samples were used to determine gene expression in the mucosa compared to protein expression that was determined by immunohistochemistry from surgical specimens with severe UC activity in all colonic layers.

These findings suggest that TRPV1 might play an important role in the pathogenesis of severe UC due to TRPV1 that was found to be overexpressed in all intestinal layers and was associated with clinical outcomes. This TRPV1 protein expression increased in all colonic layers from patients with severe active UC suggesting that upregulation is a possible defense mechanism in the colon in order to decrease bacterial invasion and inflammatory process.

UC is initiated by abnormal immune-mediated responses, mainly by CD4^+^ T cells to luminal microbial products and loss of peripheral tolerance in genetically susceptible individuals [13, 14]. CD is characterized as a Th1-mediated inflammatory response with overproduction of interferon-*γ* (IFN-*γ*) and TNF-*α* whereas UC is considered a Th2-mediated immune disease with massive production of interleukin IL-4, IL-5, IL-9, and IL-13 [15]. Few papers have shown the role of TRPV family members as ion channels functionally expressed in neuronal tissue and immune cells including T cells [15, 16]. The TRPV1 channel acts as a homotetramer and heterotetramer with other TRP channel subunits [17]. Bertin et al. demonstrated that the TRPV1 channel was expressed in CD4^+^ T cells and increased the proinflammatory profile in murine models of IBD [18]. Mechanistically, it has been identified that TRPV1 contributes to T cell receptor- (TCR-) induced calcium (Ca_2_^+^) influx and produces phosphorylation by the lymphocyte tyrosine-protein kinase, a possible gating mechanism for the TRPV1 channel in CD4^+^ T cells upon TCR stimulation [19].

Massa et al. demonstrated that TRPV1 might have a protective role in IBD and other diseases such as hypertension and sepsis [8]. In the case of gastrointestinal diseases like IBD and IBS, it has been associated with a severe inflammatory response as it was found in the present study and also reported in chronic cough and arthritis [20–23].

The present study demonstrated that TRPV1 gene expression levels negatively correlated with the degree of inflammation according to IL-6 gene expression as previously reported by De Fontgalland et al. [24] where a paradoxical decrease of TRPV1 was found in the group of patients with active UC compared to normal controls.

The protein expression analysis showed that TRPV1 was importantly increased in severe active UC patients suggesting that this upregulation of TRPV1 in the protein expression of patients with severe active UC may affect nerve terminals and immune cells suggesting that TRPV1 may have an important participation in the colonic immunomodulation of the inflammatory response as well as in the pathogenesis of UC as demonstrated in a previous study [25].

By the way, Kun et al. recently reported a strong TRPV1 immunopositivity within mononuclear and plasma cells infiltrating the colonic mucosa in patients with IBD [26]. Our study also confirmed these findings where there is an increased infiltration of TRPV1+ cells with morphology suggestive of mononuclear, plasma, and T cells in the colonic mucosa of patients with severe UC. Collectively, these results suggest that high protein expression and infiltration of TRPV1-expressing cells in the colon may contribute to the pathophysiology of IBD as also reported by Akbar et al. [25].

## 5. Conclusion

A high protein expression of TRPV1 by lymphocytes and mast, endothelial, epithelial, and muscle cells was found in severe activity of UC patients suggesting the role of TRPV1 in the development of inflammatory process in UC patients. The presence of TRPV1 gene expression was associated with young age at diagnosis and clinical course characterized by relapsing clinical course of disease in UC patients.

## Figures and Tables

**Table 1:** Oligonucleotides used for gene expression.

Gene	Left	Right
TRPV1	cagcagcgagacccctaa	Cctgcaggagtcggttca
IL-6	caggagcccagctatgaact	gaaggcagcaggcaacac
*β*-Actin	aaggcatttacttcaaacttgtca	tggattcatcagctgcattt

**Table 2:** Demographical and clinical characteristics of patients with UC.

Clinical characteristics of UC patients		*n*	%	TRPV1 expression median (range)	*P*
Gender	Male	17	50	10.53 (7.86–18.57)	0.94
	Female	17	50	10.73 (7.82–17.57)	
Age at diagnosis	<40	25	73.52	10.90 (7.82–13.29)	0.02
	>40	8	23.52	9.82 (8.18–11.37)	
Extraintestinal manifestations	Present	33	62.3	10.33 (7.83–18.57)	0.18
	Absent	20	37.7	10.93 (7.82–13.29)	
Extent of disease	Left colitis (E2) and distal colitis (E1)	15	45.5	10.73 (8.47–1.39)	0.91
	Extensive colitis (E3)	18	54.5	10.65 (7.82–13.29)	
Years of evolution	<3	18	54.5	10.65 (8.47–13.29)	1.00
	>3	15	45.5	10.90 (7.82–12.39)	
Clinical course of disease	Nonrelapsing disease	14	42.4	10.33 (7.82–13.29)	0.07
	Relapsing disease	19	57.6	10.90 (8.18–12.74)	

^∗^Extent of disease is presented according to Montreal classification for disease extent in patients with ulcerative colitis (Satsangi J. 2006).

## Data Availability

The data used to support the findings of this study are available from the corresponding author upon request.
